# Mechanisms of ventilatory acclimatization to high altitude

**DOI:** 10.1093/nsr/nwaf244

**Published:** 2025-06-13

**Authors:** Martin Burtscher, Axel Kleinsasser, Johannes Burtscher, Erik R Swenson

**Affiliations:** Department of Sport Science, University of Innsbruck, Austria; Department of Anaesthesiology and Critical Care Medicine, Medical University of Innsbruck, Austria; Department of Sport Science, University of Innsbruck, Austria; Department of Medicine, Geisel School of Medicine at Dartmouth, USA

We read with great interest the article entitled ‘Closing the loop: autonomous intelligent control for hypoxia pre-acclimatization and high-altitude health management’, recently published by Dr. Shi and colleagues [1]. They correctly emphasize that we are still far from being able to conduct optimal individualized pre-acclimatization in normobaric hypoxic conditions before climbing high mountains. Accordingly, the use of closed-loop control systems is highly promising. However, Shi *et al.* primarily discuss physiological parameters such as peripheral oxygen saturation (SpO_2_) and heart rate (HR) as critical factors for personalized closed-loop pre-acclimatization [1]. These parameters are certainly clinically relevant and can be easily recorded by wearable sensors, but they do not provide sufficient information about the individual's acclimatization status. For this, at least the additional determination of partial pressure of carbon dioxide in alveolar air (P_A_CO_2_) or arterial blood (PaCO_2_) would be required.

The complex process of acclimatization involves intricately interconnected molecular and physiological mechanisms following differing time courses [2]. The gradual increase in pulmonary ventilation (ventilatory acclimatization to hypoxia, VAH) is one of the most important mechanisms counteracting the drop in SpO_2_ during acute exposure to high altitude or hypoxia [3]. In this sense, altitude-dependent changes in SpO_2_ play an important role in the assessment of acclimatization. However, SpO_2_ recovery during acclimatization is only part of the story. Peripheral chemoreceptors (residing mainly in the carotid bodies) stimulate respiratory activity in hypoxia and are therefore responsible for the increase in P_A_O_2_, PaO_2_ and the associated SpO_2_. At the same time, hyperventilation causes a decrease in the P_A_CO_2_ and PaCO_2_, which in turn blunts the breathing mediated by central chemoreceptors located on the ventral brain stem medullary surface [4,5] and the full response of the peripheral chemoreceptors to hypoxia. When VAH is completed (usually after about 1 week at terrestrial high altitude), increased pulmonary ventilation and a related higher P_A_O_2_ are possible despite the lower PaCO_2_ values. This is likely a consequence of renal bicarbonate loss during acclimatization and reduced cerebrospinal fluid bicarbonate concentration. Therefore, in addition to SpO_2_, information on changes in PaCO_2_ is necessary to assess acclimatization status. Transcutaneous techniques offer suitable options [6], which can be easily integrated into closed-loop systems for monitoring and controlling the acclimatization status of individuals undergoing pre-acclimatization prior to high altitude trekking or climbing.
